# A Facial Solitary Vellus Hair Cyst: A Rare Entity

**DOI:** 10.7759/cureus.54159

**Published:** 2024-02-14

**Authors:** Ayushi Ghosh Moulic, Prasad Deshmukh, Sagar S Gaurkar

**Affiliations:** 1 Otolaryngology, Jawaharlal Nehru Medical College, Datta Meghe Institute of Higher Education and Research, Wardha, IND; 2 Head and Neck Surgery, Jawaharlal Nehru Medical College, Datta Meghe Institute of Higher Education and Research, Wardha, IND

**Keywords:** unusual facial location, histopathological examination, imaging studies, surgical excision, recurrent nodular swelling, vellus hair cyst

## Abstract

This case report details a rare occurrence of a vellus hair cyst presenting as a recurrent nodular swelling on the medial aspect of the right eye in a 23-year-old male. The patient underwent surgical excision guided by imaging studies, and the subsequent two-month follow-up revealed no signs of recurrence. Imaging, including contrast-enhanced computer tomography (CECT), played a crucial role in assessing the extent of the lesion and ruling out intracranial involvement. Histopathological examination confirmed the diagnosis, revealing cystic spaces with an attenuated lining containing vellus hair and marked fibrosis. The case underscores the importance of considering uncommon entities in differential diagnoses, emphasizes the efficacy of complete cyst removal in preventing recurrence, and contributes to the evolving understanding of vellus hair cysts. Further research is warranted to enhance our knowledge of their epidemiology and optimal management strategies.

## Introduction

Vellus hair cysts, or trichofolliculomas, represent a rare category of benign skin lesions characterized by cystic spaces containing vellus hairs within the wall and lumen. These cysts typically manifest in regions with a high density of hair follicles, including the face, neck, and upper trunk [1]. While vellus hair cysts are generally asymptomatic and slow-growing, their relative rarity can contribute to diagnostic challenges, especially in atypical anatomical locations [2]. The etiology of vellus hair cysts still needs to be better understood, and their infrequent occurrence has led to limited comprehensive studies on their prevalence and characteristics. The clinical diagnosis of these cysts can be complicated by their resemblance to other cutaneous cystic lesions, such as sebaceous cysts, lipomas, or dermoid cysts [3].

Surgical excision is the primary therapeutic approach for vellus hair cysts, aiming to completely remove the cyst and its wall to reduce the likelihood of recurrence [4]. Imaging modalities, such as contrast-enhanced computer tomography (CECT), may be employed to assess the extent of the lesion, particularly in cases where intracranial extension is suspected [5]. In the literature, reported cases of vellus hair cysts are sporadic, with limited documentation of their occurrence in distinct facial regions. Recognizing these cysts' unique clinical presentation and anatomical distribution is crucial for accurate diagnosis and appropriate management [6].

## Case presentation

A 23-year-old male presented to the outpatient department of otorhinolaryngology with a complaint of a solitary swelling on the medial aspect of his right eye, persisting for two months. The patient did not report any itching, pain, or tenderness associated with the swelling. Notably, the individual had a previous surgical intervention three months ago, following which a recurrence had occurred.

**Figure 1:**
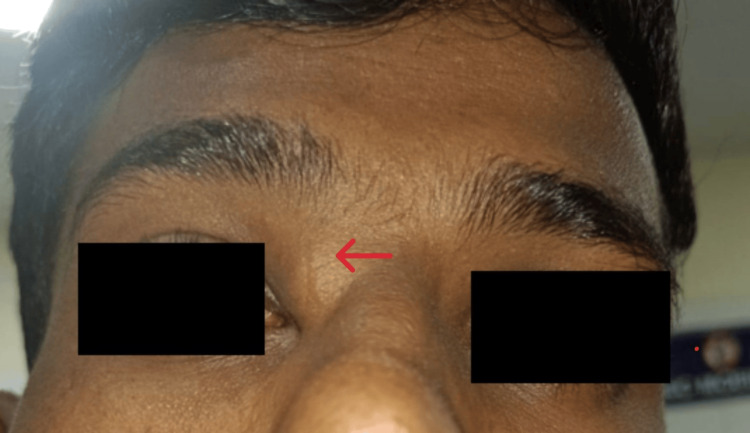
Showing a bulge over the upper part of the right medial canthus

Upon inspection, the swelling appeared as a nodular mass measuring approximately 2 cm x 2 cm, displaying an oval shape with a smooth surface. There was no evidence of discharging sinuses, and the skin color remained unchanged, as depicted in Figure 1. Palpation revealed the nodular swelling extending from the medial aspect of the right eye's inner canthus to the nose's root. It obliterated the medial canthus, demonstrated a soft consistency, and was non-fluctuant and non-tender, with no local rise in temperature.

**Figure 2:**
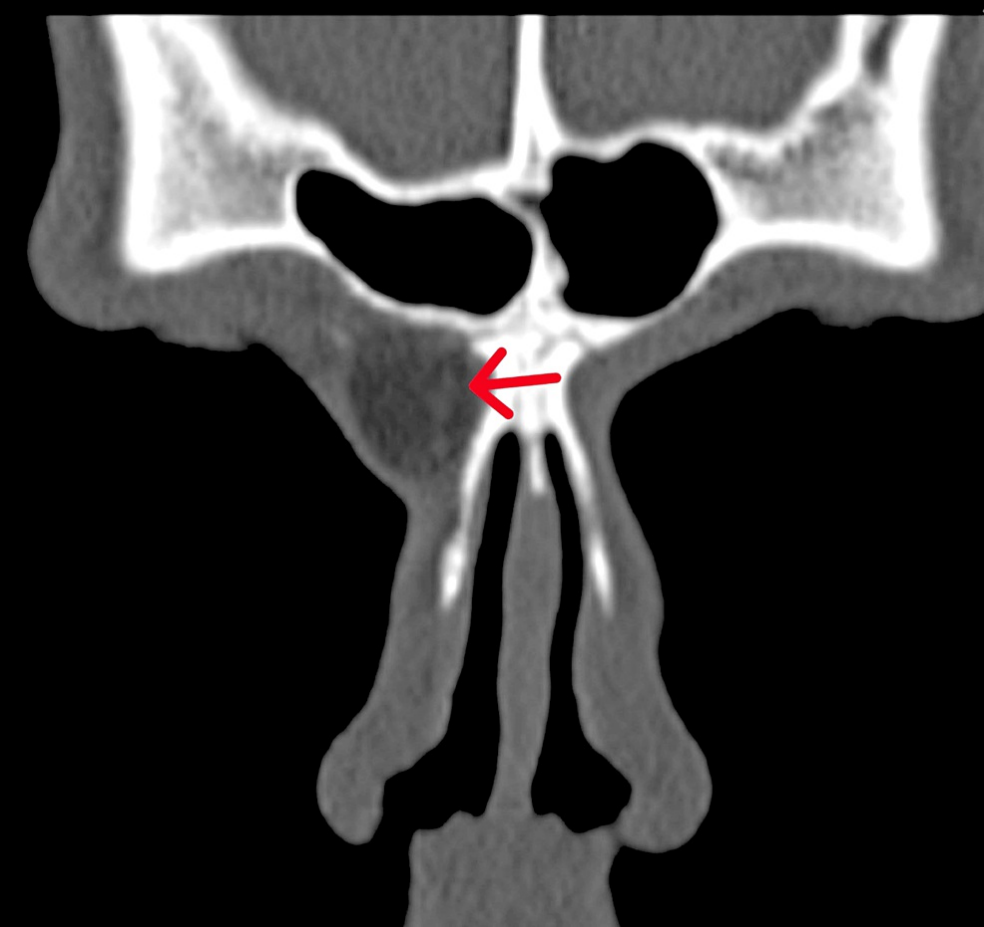
Computer tomography showing a well-defined fat density lesion in the right frontonasal region extending to anterior ethmoids

Differential diagnoses were considered, including sebaceous cyst, lipoma, and dermoid cyst. Given the unusual location of the lesion, a contrast-enhanced computer tomography (CECT) was performed to rule out any intracranial extension. The CECT revealed a well-defined lesion with fat density (-100 HU to -160 HU) in the subcutaneous plane in the right nasofrontal region, extending up to the anterior ethmoids. Mild bony erosion was observed, as illustrated in Figure 2.

**Figure 3:**
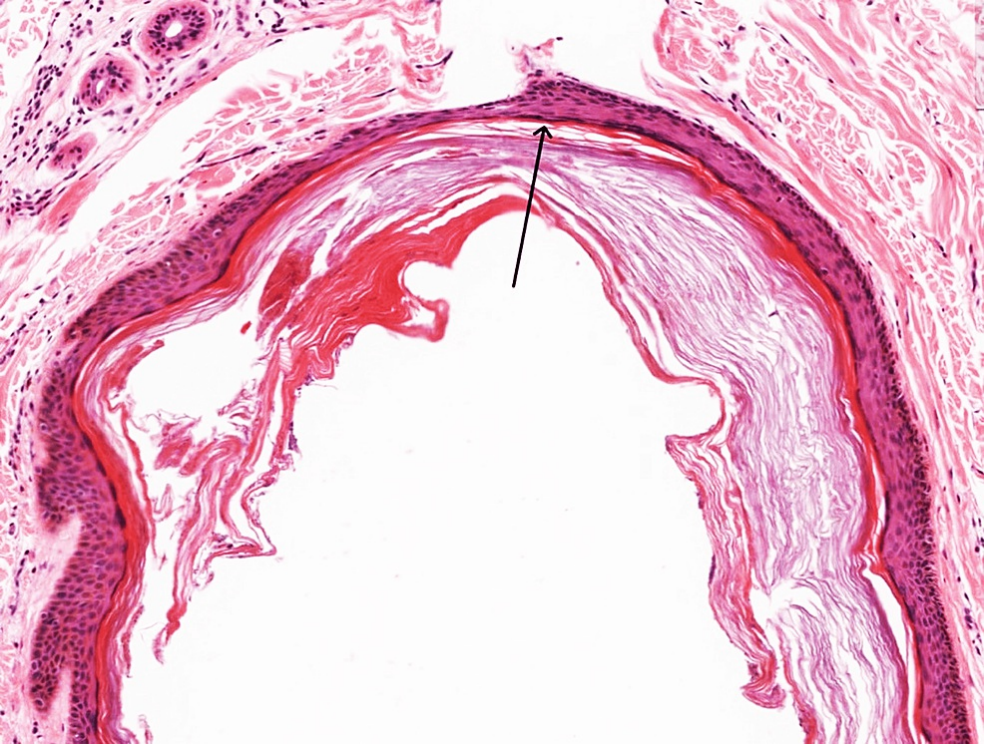
Showing a cyst line by flattened squamous epithelium with vellus hair in the wall and lumen

Subsequently, the cyst was explored, excised, and found to extend up to the right anterior ethmoids, tightly adhered to the underlying bone. A blunt dissection was conducted, and the cyst and its wall were removed and sent for histopathological examination. The wound site was closed using primary sutures. Follow-up after two months revealed complete healing with no recurrence. Histopathological examination disclosed cystic spaces with an attenuated lining containing vellus hair in both the wall and lumen. Marked fibrosis was evident, suggesting a diagnosis of vellus hair cyst, as depicted in Figure 3.

## Discussion

The presented case of a facial solitary vellus hair cyst underscores the significance of recognizing and appropriately managing rare entities in clinical practice. Vellus hair cysts are infrequently encountered, and their occurrence in the facial region, particularly the medial aspect of the eye, adds to the diagnostic complexity. This discussion will explore relevant literature, diagnostic challenges, and the optimal management of vellus hair cysts. The clinical presentation of the patient, characterized by a painless nodular swelling with a smooth surface and the absence of associated symptoms, aligns with previous reports on vellus hair cysts [2,7]. However, the rarity of this lesion in such a specific facial location highlights the importance of considering diverse differential diagnoses.

The differential diagnosis initially included a sebaceous cyst, lipoma, and dermoid cyst, in line with common lesions occurring in the head and neck region. Contrast-enhanced computer tomography (CECT) played a pivotal role in this case to rule out intracranial extension and evaluate the extent of the lesion [8]. The imaging findings, such as fat density and mild bony erosion, were consistent with literature describing vellus hair cysts, aiding in confirming the diagnosis [9]. Surgical excision remains the mainstay of treatment for vellus hair cysts, as demonstrated in this case. Complete removal is crucial to prevent recurrence, as incomplete excision can lead to the persistence or regrowth of the cyst [10]. The surgical procedure involved exploration, blunt dissection, and removal of the cyst along with its wall. The absence of recurrence during the two-month follow-up period indicates the success of the intervention.

The histopathological examination confirmed the vellus hair cyst, revealing cystic spaces with an attenuated lining containing vellus hair and marked fibrosis. This histological pattern aligns with previous descriptions of vellus hair cysts, further validating the diagnosis [11]. While vellus hair cysts are generally benign, their unusual locations may pose diagnostic challenges, emphasizing the need for a multidisciplinary approach involving clinical, radiological, and pathological assessments. Awareness of the entity and its clinical manifestations is crucial for accurate diagnosis and appropriate management.

## Conclusions

In conclusion, the presented case of a vellus hair cyst in a rare facial location underscores the diagnostic challenges and therapeutic considerations associated with this uncommon dermatological entity. The successful surgical excision, guided by imaging studies and comprehensive clinical evaluation, prevented recurrence during the two-month follow-up period. Histopathological analysis was crucial in confirming the diagnosis, revealing characteristic features of vellus hair cysts, including cystic spaces with an attenuated lining containing vellus hair and marked fibrosis. This case contributes to the limited literature on vellus hair cysts, emphasizing the need for heightened clinical awareness, accurate diagnosis, and meticulous surgical management for optimal patient outcomes. Further research is warranted to enhance our understanding of the epidemiology and pathogenesis of vellus hair cysts, enabling the development of standardized diagnostic and therapeutic approaches for these rare cutaneous lesions.

## References

[1] Patokar AS, Holani AR, Khandait GH, Khatu SS (2022). Eruptive vellus hair cysts: an underdiagnosed entity. Int J Trichology.

[2] Khatu S, Vasani R, Amin S (2013). Eruptive vellus hair cyst presenting as asymptomatic follicular papules on extremities. Indian Dermatol Online J.

[3] Espinoza Hernández CJ, Fonte Ávalos V (2013). Eruptive vellus hair cysts: prevalence and clinical features [Article in Spanish]. Gac Med Mex.

[4] Shukla R, Karagaiah P, Patil A (2022). Surgical treatment in hidradenitis suppurativa. J Clin Med.

[5] Vishwanath V, Jafarieh S, Rembielak A (2020). The role of imaging in head and neck cancer: an overview of different imaging modalities in primary diagnosis and staging of the disease. J Contemp Brachytherapy.

[6] Waldemer-Streyer RJ, Jacobsen E (2017). A tale of two cysts: steatocystoma multiplex and eruptive vellus hair cysts-two case reports and a review of the literature. Case Rep Dermatol Med.

[7] Torchia D, Vega J, Schachner LA (2012). Eruptive vellus hair cysts: a systematic review. Am J Clin Dermatol.

[8] Mittal MK, Malik A, Sureka B, Thukral BB (2012). Cystic masses of neck: a pictorial review. Indian J Radiol Imaging.

[9] Kamra HT, Gadgil PA, Ovhal AG, Narkhede RR (2013). Steatocystoma multiplex-a rare genetic disorder: a case report and review of the literature. J Clin Diagn Res.

[10] Kaya TI, Tataroglu C, Tursen U, Ikizoglu G (2006). Eruptive vellus hair cysts: an effective extraction technique for treatment and diagnosis. J Eur Acad Dermatol Venereol.

[11] Anand P, Sarin N, Misri R, Khurana VK (2018). Eruptive vellus hair cyst: an uncommon and underdiagnosed entity. Int J Trichology.

